# Inducibly decreased MITF levels do not affect proliferation and phenotype switching but reduce differentiation of melanoma cells

**DOI:** 10.1111/jcmm.13506

**Published:** 2018-01-25

**Authors:** Kateřina Vlčková, Jiri Vachtenheim, Jiri Réda, Pavel Horák, Lubica Ondrušová

**Affiliations:** ^1^ Department of Transcription and Cell Signaling Institute of Medical Biochemistry and Laboratory Diagnostics First Faculty of Medicine Charles University Prague Czech Republic

**Keywords:** melanoma, MITF, phenotype switching, proliferation, invasiveness, differentiation

## Abstract

Melanoma arises from neural crest‐derived melanocytes which reside mostly in the skin in an adult organism. Epithelial–mesenchymal transition (EMT) is a tumorigenic programme through which cells acquire mesenchymal, more pro‐oncogenic phenotype. The reversible phenotype switching is an event still not completely understood in melanoma. The EMT features and increased invasiveness are associated with lower levels of the pivotal lineage identity maintaining and melanoma‐specific transcription factor MITF (microphthalmia‐associated transcription factor), whereas increased proliferation is linked to higher MITF levels. However, the precise role of MITF in phenotype switching is still loosely characterized. To exclude the changes occurring upstream of MITF during MITF regulation *in vivo*, we employed a model whereby MITF expression was inducibly regulated by shRNA in melanoma cell lines. We found that the decrease in MITF caused only moderate attenuation of proliferation of the whole cell line population. Proliferation was decreased in five of 15 isolated clones, in three of them profoundly. Reduction in MITF levels alone did not generally produce EMT‐like characteristics. The stem cell marker levels also did not change appreciably, only a sharp increase in SOX2 accompanied MITF down‐regulation. Oppositely, the downstream differentiation markers and the MITF transcriptional targets melastatin and tyrosinase were profoundly decreased, as well as the downstream target livin. Surprisingly, after the MITF decline, invasiveness was not appreciably affected, independently of proliferation. The results suggest that low levels of MITF may still maintain relatively high proliferation and might reflect, rather than cause, the EMT‐like changes occurring in melanoma.

## Introduction

Malignant melanoma is an aggressive tumour of neuroectodermal origin that has a dismal prognosis if it is not excised at an early stage. More than 50% of melanoma cases harbour the BRAF(V600E) mutation 1, 2. However, singular targeted inhibition of BRAF leads invariably to acquired resistance (which can be also inherent) that can result in worsening of the patient's prognosis also through inducing the therapy‐induced pro‐oncogenic secretome 3. Melanoma cells are very early phenotypically diversified and undergo phenotype switching resembling the EMT, through which they acquire considerable microheterogeneity resulting in plasticity, capability of invasion and migration. These properties lead to metastasis and poor prognosis 4, 5, 6, 7. EMT is mostly a reversible process through which undergoes epithelial tumours to gain the mesenchymal phenotype and more oncogenic characteristics but occurs also in non‐epithelial cancers 8, 9.

Melanocyte‐specific isoform of MITF (microphthalmia‐associated transcription factor) is a pivotal protein determining the melanocyte lineage identity and conferring a strong antiapoptotic activity to melanoma cells 10. This is accomplished through the direct activation of expression of several antiapoptosis factors such as BCL2 11, livin 12, BPTF 13 and others.

Two phenotypically distinct populations of melanoma cells were described related to MITF levels: High‐MITF population is associated with differentiation and proliferation, whereas low‐MITF cells, although they proliferate slowly, are endowed with the invasive and EMT‐like characteristics 14, and they express pro‐oncogenic genes such as Brn2 15, 16, 17, 18, 19, GLI2 20, JARID1B 21, Axl 22 and others. On the other hand, it has been found that a large proportion (over 25%) of melanoma cells derived directly from patients are capable of forming tumours in highly immunocompromised NOD/SCID interleukin‐2 receptor gamma chain null (Il2rg(−/−), NSG) mice 23. Also, the phenotypic heterogeneity in melanoma is extremely reversible and not hierarchically organized 24. These findings substantially challenge the concept of a small population of rare cancer initiating cells with stem cell (SC) properties 21, 25, 26 which are recruited from the invasive cells and have a high self‐renewal potential and propensity to form metastasis.

When studying the phenotypic changes in melanoma, it is crucial to discern the effects of MITF alone from the effects of expression changes in many MITF transcriptional regulators and cofactors that operate upstream of MITF. They undoubtedly influence not only MITF but also many other targets involved in the phenotype outcome *in vivo*. Events caused purely by MITF down‐regulation can be achieved through manipulating MITF levels alone, an approach that is not feasible to perform *in vivo*. It is thus highly desirable to understand precisely the mechanisms which MITF plays in modulating tumour cell invasiveness, plasticity, migration, proliferation and metastasis *in vitro* and *in vivo*.

We used here the doxycycline (DOX)‐based inducible lentiviral system to stepwise decrease MITF level in six melanoma cell lines. In this setting, the expression of upstream genes regulating MITF expression remained intact, simplifying the interpretation of phenotype changes and evaluation of the effect of exclusive down‐regulation of MITF. We found no profound changes in proliferation of whole cell populations, EMT gene expression pattern and invasiveness. In contrast, the expression of the downstream differentiation markers melastatin and tyrosinase and the antiapoptotic MITF target livin diminished after DOX‐dependent reduction in MITF protein level. Based on these experiments with cell lines, we suggest slightly modified model concerning the role of MITF in proliferation and invasiveness of melanoma cells. The data further suggest that more complex events may occur during the phenotype switching in melanoma that might be a more non‐uniform process than previously anticipated and may be a cause (rather than a result of) of the low‐MITF levels in the invasive subpopulations.

## Materials and methods

### Cell culture

Melanoma cell lines SK‐MEL‐3, SK‐MEL‐5, SK‐MEL‐28, Malme 3M and MeWo were purchased from ATCC and were grown using EMEM complete medium with non‐essential amino acids and pyruvate, or RPMI1640 medium (for Malme 3M). 501mel cells were generously provided by Dr. R. Halaban (Yale University) and maintained in RPMI1640 medium. All media were supplemented with 10% FCS and antibiotics. All cell lines harbour mutated BRAF(V600E), with the exception of MeWo cells which are BRAFwt; 293FT cells were purchased from Invitrogen (Carlsbad, CA, USA) and cultivated in DMEM with 10% FCS.

### Proliferation assays

#### Colony outgrowth assay

After culturing the cells 6 days in appropriate concentration of DOX (Invitrogen), cells were seeded at low density in 12‐well plates and grown for 9 days. The medium with or without (as a control) DOX was changed every other day. Cells were then fixed, stained with crystal violet and quantified.

#### Growth curves

This experiment reflects the cell growth after previous long‐term cultivation in DOX. Cells were first maintained for 5 weeks in appropriate DOX concentration, then plated in 24‐well plates at low density and fixed on days 0, 3, 6 and 9. Medium was changed every other day. Cells were fixed, stained with crystal violet, destained and quantitated on a spectrophotometer. Growth curves were constructed using the triplicate data. The levels of MITF in DOX remained decreased all the time as assessed by Western blot. Curves are shown with a standard error for each point.

### Western blot analysis and immunofluorescence

Cells were lysed in a complete RIPA buffer (1% NP‐40, 150 mM NaCl, 5 mM EDTA, 0.5% sodium deoxycholate, 50 mM Tris‐HCl pH 7.5, 0.1% SDS) with added protease and phosphatase inhibitors aprotinin, leupeptin, pepstatin, phenylmethylsulphonylfluoride and PhosStop (Roche, Indianapolis, IN, USA). After the electrophoresis on 10–12% SDS–polyacrylamide gels, the proteins were transferred onto PVDF membrane (Millipore, Billerica, MA, USA). Blots were incubated with primary and horseradish peroxidase‐conjugated secondary antibodies and detected by chemiluminescent determination. The following commercially available antibodies were used for Western blots: antibody against MITF (cat. no. MS‐772; Neomarkers, Fremont, CA, USA), BCL2 (556 354; Becton Dickinson, San Diego, CA, USA), livin (sc‐30161; Santa Cruz Biotechnology, Dallas, TX, USA), Axl (sc‐166269; Santa Cruz), β‐catenin (8480; Cell Signaling, Danvers, MA, USA), SRC (2109; Cell Signaling), β‐actin (A5316; Sigma‐Aldrich, St Louis, MO, USA), E‐cadherin (3195; Cell Signaling), N‐cadherin (13116; Cell Signaling), SLUG (9585; Cell Signaling), SNAIL Santa Cruz, sc‐28199), vimentin (5741; Cell Signaling), ZEB1 (3396; Cell Signaling), ZEB2 (sc‐271984; Santa Cruz), p27 (Santa Cruz, sc‐528), KLF4 (LS‐C415468; LSBiotechnologies, Seattle, WA, USA), ALDH1A1 (LS‐B10149; LSBiotechnologies), Brn2 (sc‐393324; Santa Cruz), SOX2 (5024; Cell Signaling), OCT4 (sc‐514295; Santa Cruz). For immunofluorescence, cells were fixed in 3% paraformaldehyde the next day after seeding, permeabilized and stained with anti‐MITF antibody followed by a FITC‐labelled second antibody. Cell chambers were then mounted in the mounting medium with DAPI.

### Lentivirus production and infection of target cells

ShRNA‐coding hairpin sequence against MITF 27 was cloned in DOX‐inducible (Tet‐On) Tet‐pLKO‐puro plasmid 28 (Addgene plasmid no. 21915). This shRNA sequence has been previously verified and down‐regulates MITF level best among other tested sequences. Lentiviruses were packaged in 293FT cells as described earlier 29. Plasmid with scrambled shRNA sequence was used as a control. Six melanoma cell lines (above) were infected with the fresh virus overnight in the presence of 6 μg/ml Polybrene (Sigma‐Aldrich) and then briefly (4–5 days) selected in puromycin (Sigma‐Aldrich) and maintained in low puromycin (0.25 μg/ml) media.

### Invasivity and wound‐healing assay

For these assays, cells were grown for 6 days in medium without DOX and with 1 μg/ml DOX (or in 0.5 μg/ml DOX for wound‐healing assay). Estimation of cell invasiveness has been made using the collagen invasivity kit (Millipore). For the wound scratch migration assays, cells were prepared in duplicates on 12‐well plates. Next day, cells were near‐confluent and wounded using 1‐ml sterile pipette tip and photodocumented for control time zero, washed repeatedly and starved for 24 hrs in medium containing 0.5% FCS. Next day, cultivation medium containing 15% FCS was added (still keeping the cells with or without DOX), and invasion of cells in the same areas as at the time zero was photodocumented after next 24 and 48 hrs.

### Viability

Cell viability was estimated on cells in duplicates. Cells bearing the inducible shRNA against MITF or control cells were treated for 6 days with the indicated concentrations of DOX, replated onto 12‐well plates, and viability was determined next day by the MTT viability kit (Sigma‐Aldrich) according to the manufacturer's instructions.

### Real‐time PCR

Estimation of melastatin mRNA levels was performed with primers and a labelled probe as described in the original procedure 12. Primers for estimation of tyrosinase were as follows: forward, 5′‐ CCAGAAGCTGACAGGAGATG; reverse, 5′‐ AGGCATTGTGCATGCTGCTT; probe, 5′‐FAM‐ACGGCGTAATCCTGGAAACCATGACA‐TAMRA. After total RNA was isolated using TRIzol (Life Technologies, Carlsbad, CA, USA), 2 μg of RNA was reverse‐transcribed using transcriptor reverse transcriptase (Roche), and cDNAs for melastatin and tyrosinase were quantitated using Taqman system QuantiTect Probe PCR Kit (Qiagen, Hilden, Germany). Data were acquired on a ViiA7 system (Life Technologies). Each experiment has been performed twice with similar results. Data are presented after the compensation to β‐actin mRNA levels as a control gene.

### Statistics

Each experiment was performed at least two times with consistent results. Data in graphs are presented as means and their standard errors. Statistical significance was determined using the Student's *t*‐test. *P* value <0.05 or <0.01 was considered statistically significant as indicated. For quantification of proliferation assays, the ImageJ software (National Institutes of Health, Bethesda, MD, USA) was employed, and one of two experiments is presented.

## Results

### Reduced MITF levels do not cause halt of proliferation or cell cycle arrest

**Figure 1:**
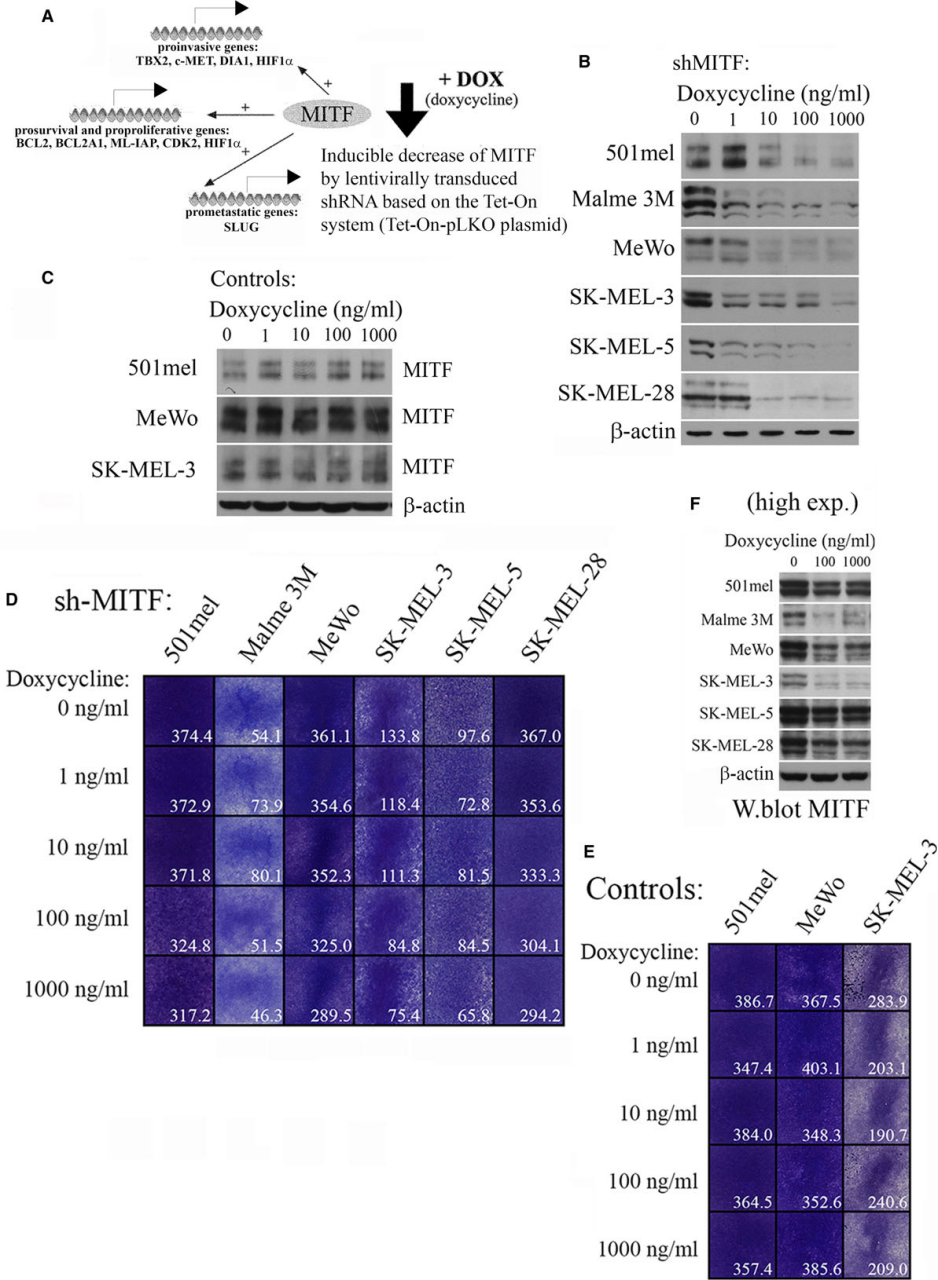
Gradually decreased MITF protein in the inducible system causes minimal changes in proliferation. (**A**) A scheme of experimental setting with a view of groups of MITF‐inducible genes. (**B**) Infection of six melanoma cell lines with a lentivirus carrying the shRNA sequence directed to MITF, followed by a brief puromycin selection. Incubation of cells in increasing concentrations of DOX leads to a stepwise disappearance of the MITF Western blot signal. The Western blot was performed 6 days after incubation without or with DOX. (**C**) No MITF level changes were seen in control virus‐infected cultures. (**D**) Proliferation rates are determined in increasing DOX concentrations. Cells were maintained in DOX for 6 days, and then, the experiment was carried out and quantitated by ImageJ. Two experiments with consistent results were performed and one is presented. (**E**) Similar to **D**, control virus‐infected cultures grew in all DOX concentrations. The setting of the experiment was the same as in **D**. (**E**) Longer exposure of the same Western blots as shown in **B**. Only two highest DOX concentrations are shown. In all cell lines, some residual MITF remains even in the highest DOX concentration.

We generated lentivirus encoding shRNA‐MITF enabling the regulatable decrease in MITF levels in cell lines. This approach enables the elimination of MITF upstream events that occur *in vivo*, which down‐regulate MITF but can have many other activities which are MITF‐independent. Thus, in our system, only MITF‐regulated genes are participating in the resulting phenotype (schematic Fig. 1A).

Cells infected with the Tet‐pLKO‐puro‐based produced virus were selected in puromycin and constituted DOX‐responsive cell lines with gradually decreased MITF levels after increasing DOX doses. Six cell lines with high or average MITF levels were chosen to better follow the stepwise MITF depletion. We used 1 μg/ml of DOX as the highest concentration because higher DOX began to cause a non‐specific toxic effect to the cells. MITF levels decreased gradually with increasing DOX doses in all cell lines tested (Fig. 1B). No change in MITF level was seen in control virus‐infected cells, as exemplified in three cell lines (Fig. 1C). Although MITF protein was substantially decreased (Fig. 1B), high exposures revealed still appreciable levels even in 1 μg/ml of DOX (Fig. 1F). This is in contrast with our previous results where we were able to ablate MITF completely (targeting the same sequence) with transfected non‐inducible pSUPER‐puro‐shMITF plasmid and puromycin selection 27 in 501mel cells (Fig. S1), which was followed by cell cycle arrest and subsequent apoptosis. The difference in results is apparently due to the different silencing system and a very effective block of MITF expression when shRNA was cloned in pSUPER plasmid and transfected. To substantiate the knockdown, we verified the decreased MITF levels and assessed the results by immunofluorescence. MITF staining was decreased in all DOX‐treated cells but not in controls (Fig. S2).

Surprisingly, even the highest decrease in MITF had relatively little effect on cell proliferation in this study, as assessed by colony formation assay (Fig. 1D). Evidently, a smaller decrease in proliferation was seen in most cell lines at high DOX, but a very slight retardation of growth was visible also in some controls (Fig. 1E). Collectively, reduction in MITF levels had no dramatic effect on proliferation rate in melanoma cell lines, probably partly because the degree of knockdown left some MITF level which was sufficient for proliferation. Consistent with this, little or no changes in the cell cycle profiles were observed in cells without DOX or containing 1 μg/ml of DOX (Fig. S3). The data thus show that even small amounts of MITF are capable of maintaining proliferation of melanoma cells.

**Figure 2:**
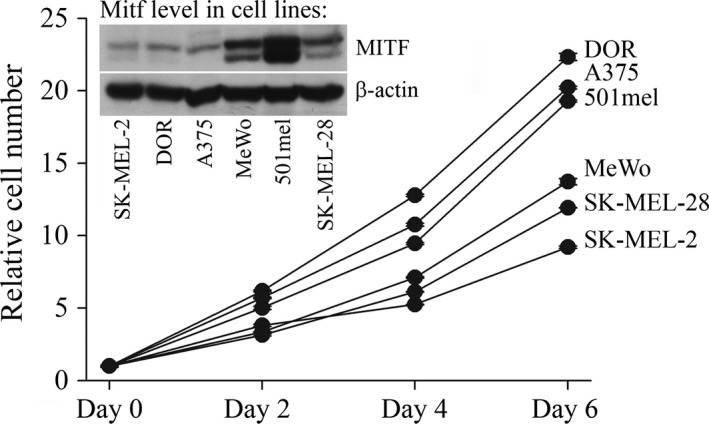
The proliferation rates of native three low‐MITF and three high‐MITF melanoma cell lines are completely intermingled. Cells were seeded at lower density in triplicates and were fixed every other day. There were only minimal changes among triplicates, as demonstrated by extremely small S.E. values. Insert: Western blot stained with the anti‐MITF antibody shows the MITF protein levels in cell lines analysed and equal loading (β‐actin).

To test the growth of established melanoma lines, we chose three low‐MITF and three high‐MITF cell lines and determined their proliferation rate. There was no relationship between the MITF level and proliferation (Fig. 2). The growth rate data of low‐MITF cells (SK‐MEL‐2, Dor and A375) were completely mixed with the data of high‐MITF cell lines (MeWo, 501mel, SK‐MEL‐28), indicating that even low level of MITF can sustain high proliferation rate in some melanomas, apparently dependent on the cellular context.

### Inducible reduction in MITF protein generally does not induce the phenotype switching towards EMT changes or expression of SC markers

**Figure 3:**
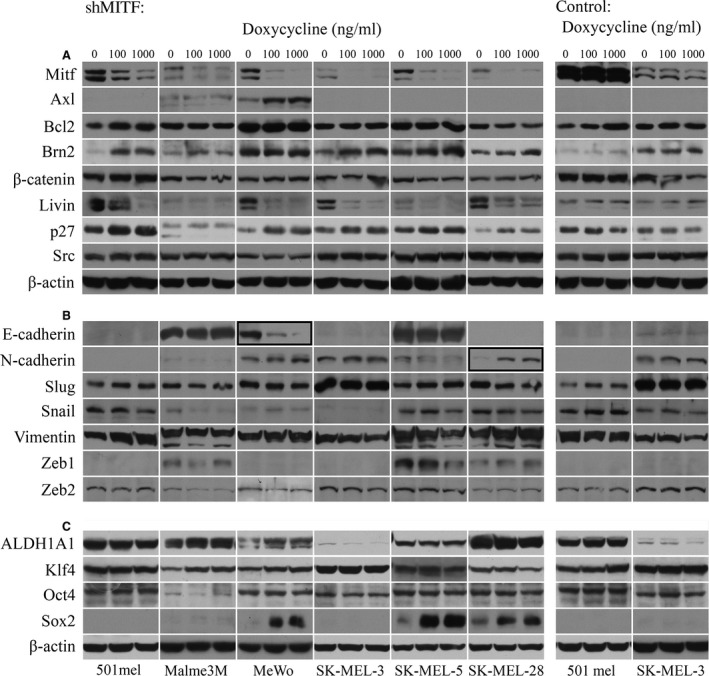
Pattern of gene expression after down‐regulating MITF by two highest DOX concentrations. (**A**) Western blot of proteins not directly connected with the EMT‐like process. Livin is a MITF target and mirrors the decrease appearing in MITF samples. Axl is negatively correlated with MITF only in MeWo (the only BRAFwt cell line), where it is most prominently expressed. Actin control shows equal loading. (**B**) The proteins which are often associated with EMT. Two triplets of typical EMT changes (E‐cadherin in MeWo and N‐cadherin in SK‐MEL‐28) are framed. Loading and sample's integrity are demonstrated by expression by SLUG and vimentin expression. (**C**) Stem cell markers expression. Two control virus‐infected cell lines are also shown (right). Some proteins (*e.g*. Axl or SOX2) were expressed only in some cell lines. All cells were maintained in DOX for 6 days before performing the Western blots.

Although MITF levels are not critical for proliferation either in an artificial inducible system or in native cell lines (above), the presence of MITF is essential to prevent apoptosis in melanoma cells 10, 27, 30. Furthermore, low‐MITF populations of cells are believed to proliferate slowly but to be highly invasive, while high‐MITF cells are proliferating rapidly and are not invasive. This ‘rheostat model’ has been proposed first in 501mel cells 14. As invasive cells undergo EMT‐like changes, we studied whether the inducible MITF decrease *per se* could induce EMT hallmarks. The EMT‐like changes in melanoma are characterized by the increased expression of markers such as SNAIL, ZEB1, N‐cadherin, vimentin and decreased E‐cadherin 31, 32. We first determined expression levels of proteins previously reported to be important for melanoma progression (Fig. 3A). We found no change in SRC and β‐actin as controls. Also β‐catenin did not display any changes. BCL2, a MITF target, did not decrease as well (only slightly in SK‐MEL‐28). Axl level increased in MeWo but remained unchanged in Malme 3M upon DOX addition and was not present in other cell lines. On the other hand, livin perfectly mimicked the down‐regulation of MITF (Fig. 3A) as it is a known MITF downstream target. P27 protein was found increased after increasing DOX levels in three cell lines, very slightly increased in two lines and remained unchanged in one line (Fig. 3A). This cdk inhibitor has been originally described to be the cause of inhibition of proliferation in pro‐invasive subpopulations 14, 33. Brn2 protein appeared increased in MITF‐lowered samples in four cell lines, most prominently in 501mel cells (consistent with the original model 14), while it remained unchanged in two cell lines (Fig. 3A).

As a next step, we have estimated markers which should undergo changes during the EMT‐like process after the MITF decrease. We analysed protein levels of vimentin, E‐cadherin, N‐cadherin, SLUG, SNAIL, ZEB1 and ZEB2. ZEB2 and SLUG are mostly considered to be pro‐proliferative and pro‐differentiative markers, not involved in the EMT process in melanoma) and revealed a pattern showing only minimal changes (Fig. 3B). The only two characteristic pictures typically reflecting EMT were the decrease in E‐cadherin in MeWo cells and increase in N‐cadherin in SK‐MEL‐28 cells. Vimentin was uniformly expressed with increase in 501mel and SK‐MEL‐28 cells and a low decrease in SK‐MEL‐3 cells. Further, many EMT‐related proteins were absent from cells at all DOX concentrations (*e.g*. E‐cadherin and ZEB1 were absent in three different cell lines). In aggregate, lowering of MITF levels alone generally does not lead to EMT‐like phenotype patterns on Western blots in six melanoma cell lines.

We examined also the pattern of cancer SC markers after MITF down‐regulation. We found no change in the level of proteins ALDH1A1, KLF4 and OCT4 (Nanog was negative in all cell lines, not shown), whereas a profound increase in SOX2 was observed at both DOX concentration in SK‐MEL‐3, SK‐MEL‐5 and SK‐MEL‐28 cell lines. Other three lines did not express SOX2 (Fig. 3C). Thus, for a high expression of SC marker SOX2, which is critical for forming the tumour‐initiating cells in melanoma 34, low‐MITF level is required. This finding is consistent with the accepted model that the melanoma SC is recruited from the invasive low‐MITF populations.

### The growth rate of the whole cell population remains unchanged in low‐MITF long‐term cultures

As the proliferation assays after several days in DOX did not show any substantial growth diminution, we reasoned that longer cultivation of cells in DOX could be required to achieve the effect of more prominent growth deceleration. The cell lines were cultured for 5 weeks with or without DOX, and the proliferation curves were determined during next 9 days. The same experiment was also performed with control virus‐infected cells to exclude the possible non‐specific effect of DOX at the highest concentration. No substantial changes were observed when proliferation of pooled cultures cultivated in media –DOX and +DOX was estimated (Fig. S4A); 501mel cells +DOX ceased to grow at the end of the experiment, probably because their proliferation is highly dependent on MITF 27. Proliferation of some cell lines was slower even from the day 3 onwards, but this phenomenon was seen also in controls (MeWo and MeWo control, SK‐MEL‐3 and SK‐MEL‐5 control). Control Western blots confirmed lower MITF in DOX‐containing cultures after the long‐term cultivation (Fig. S4B). Together, the maintenance of melanoma cells in up to 1000 ng/ml DOX did not have any great deleterious effect on the rate of long‐term proliferation in pools of infected cell lines.

### In long‐term cultures, the minority of individual clones with reduced MITF reveals slow proliferation

**Figure 4:**
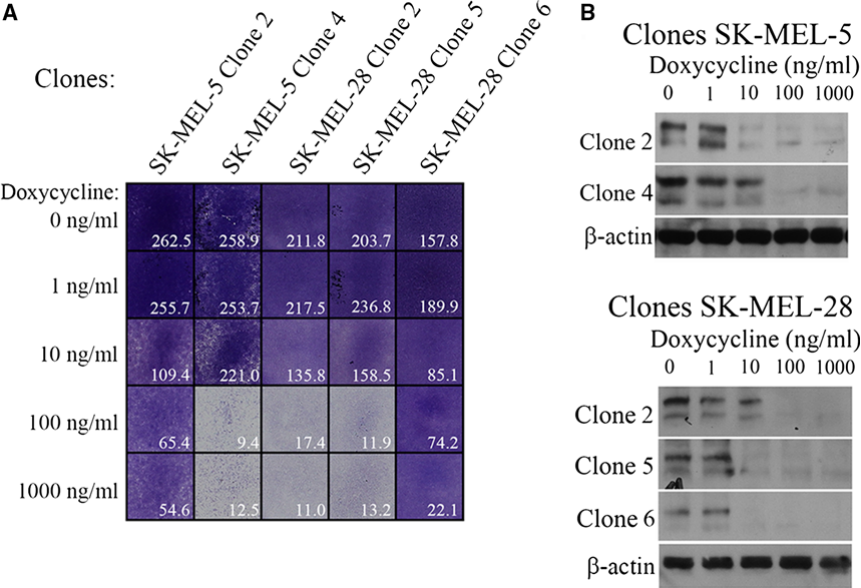
Cell proliferation of five isolated and expanded clones. (**A**) Of 15 isolated clones from cell lines SK‐MEL‐5 or SK‐MEL‐28, maintained in DOX for 4–5 weeks required for expansion, only five clones (shown) revealed prominent decrease in growth in colony outgrowth assay. Two identical experiments gave similar results, and one experiment is depicted. Other 10 clones resembled minimal proliferation changes comparable to Figure 1 **D** (not shown). Proliferation pictures were obtained during 9‐day incubation in appropriate DOX concentration. After the removal of DOX, the slowdown clones recovered to near‐to‐normal proliferation rate. (**B**) Confirmation of stepwise MITF protein diminution on Western blots after DOX treatment in the five clones used in **A**, performed at the beginning of the proliferation experiment.

Given the proliferation of the whole cell population was only slightly affected by MITF decrease, we investigated whether the growth of cultures raised from the individual cell clones could be retarded in DOX. To this end, we isolated and expanded 15 randomly chosen individual clones from SK‐MEL‐28 or SK‐MEL‐5 cell lines and maintained them in 0, 100 or 1000 ng/ml DOX concentrations for 5 weeks. The proliferation was determined thereafter by the colony outgrowth assay. We found five (of 15) clones that were substantially retarded in proliferation in these low‐MITF cultures. The three most retarded clones were two SK‐MEL‐28 clones and one SK‐MEL‐5 clone (Fig. 4A). These three expanded clones retained less than about 15% of proliferation propensity compared the normal growth of the majority of clones. Besides these, other two clones showed decreased growth rate about fourfold to fivefold (Fig. 4A). The control Western blot revealed that MITF still remained gradually decreased at the time of the experiment in these clone‐derived cultures maintained in DOX (Fig. 4B). Thus, some individual clones can indeed react to the lowered MITF by exclusive severe growth retardation. We hypothesize that this may happen by the absence of sufficient antiapoptotic signals that were probably almost entirely dependent on MITF in these clones. This experiment strengthens the enormous heterogeneity at the single cell level even in the relatively homologous cell line population.

### Invasiveness and migration are not affected by reduced MITF levels

**Figure 5:**
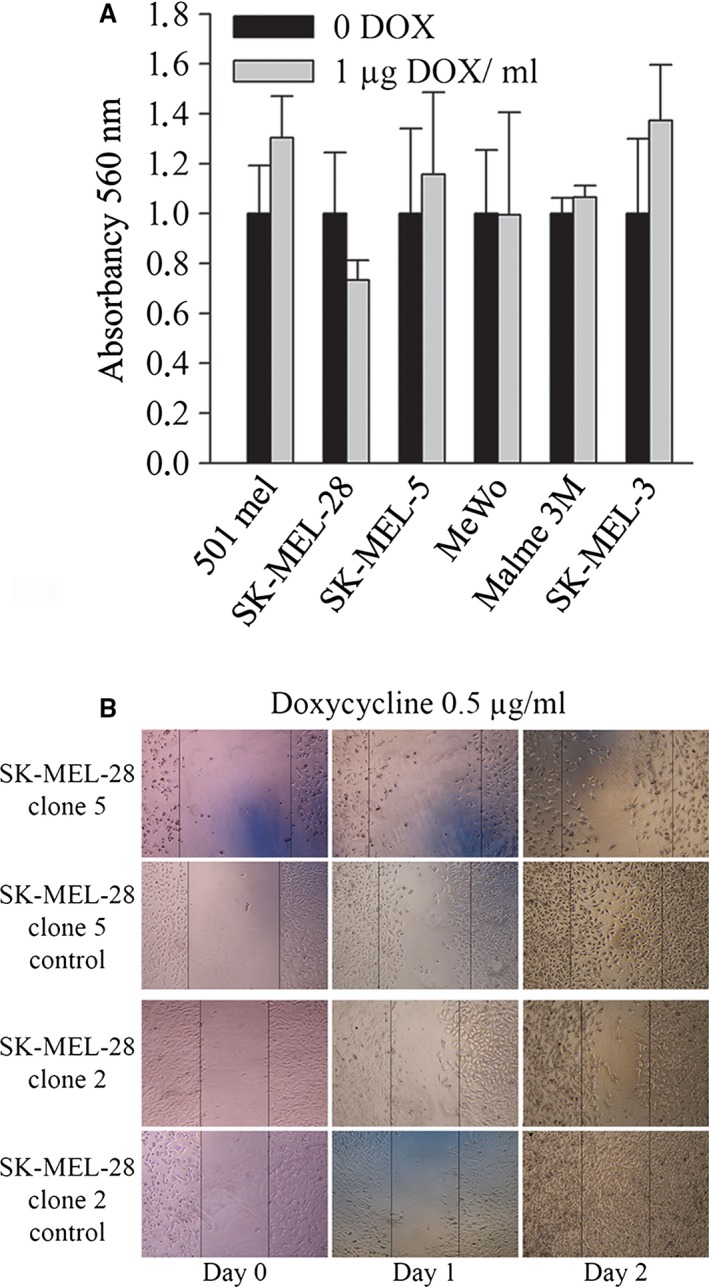
Cell invasiveness of cell lines and migration assay of selected clones. (**A**) Collagen invasivity assay of six cell lines in medium without DOX and with the highest DOX concentration 1 μg/ml, performed after 6 days in appropriate medium. All results show insignificant changes in invasiveness. Two experiments with similar results were performed and one is presented. (**B**) Wound‐healing assays of the two slowly proliferating clones. Note that migration in 0.5 μg/ml DOX is presented as concentrations 100 and 1000 nM produced similar results (not shown).

Because the low proliferation of melanoma cells has been reported to be associated with increased invasivity 14, 33, we have estimated invasiveness in the proliferating whole cell populations and in slowly proliferating clones. The collagen matrix invasion assay showed no significant changes between DOX‐treated and non‐treated cells in all cell lines (Fig. 5A) and clones (not shown). Similarly, the migration assay after cell scratches did not reveal any changes (Figs 5B and S5). Not unexpectedly, the very slow proliferation of the three clones (Fig. 4A) was accompanied with no increase in migration properties, as exemplified by the scratch assay in two clones (Fig. 5B). Next, the viability was tested in whole cell populations, and significant decrease was revealed in three cell lines (SK‐MEL‐3, SK‐MEL‐5 and SK‐MEL‐28) at high DOX concentrations (Fig. S6). This was in accord with the observation that these lines also revealed relatively higher growth retardation (Fig. 1D). These data indicate that viability was a sensitive assay for the detection of phenotype changes after lowering MITF and possibly reflects higher apoptosis in lower viable cells.

### Reduction in MITF levels decreases expression of downstream MITF differentiation markers

**Figure 6:**
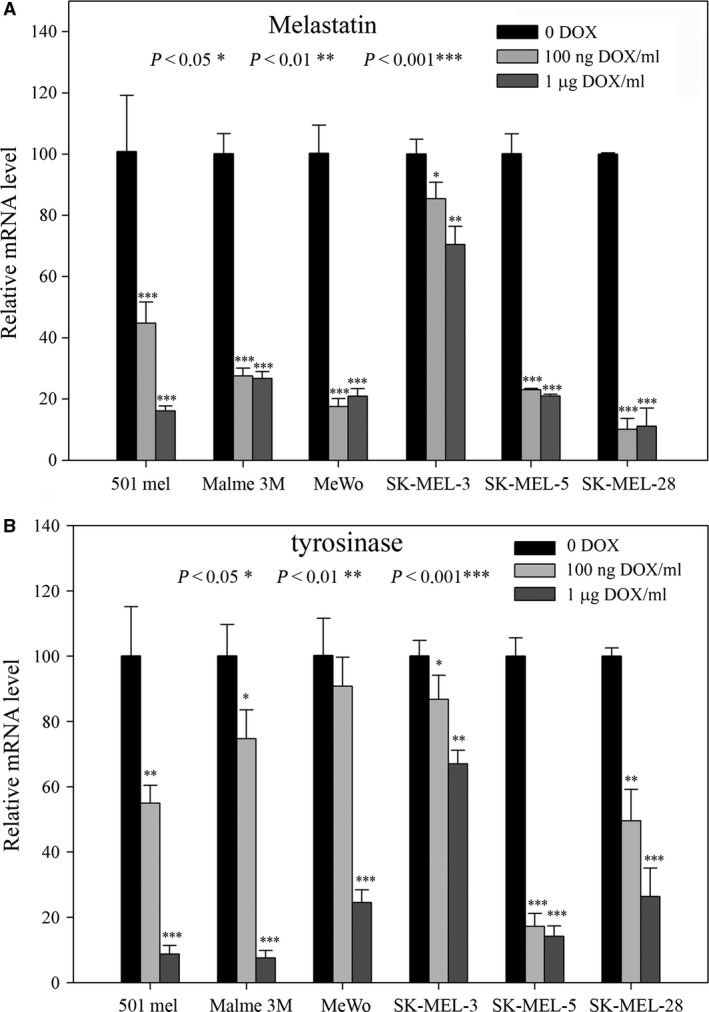
Real‐time PCR results detecting mRNA levels of MITF downstream differentiation markers. (**A**) The changes in melastatin mRNA levels after incubation of cells in DOX. (**B**) Levels of tyrosinase mRNA. All deviations from the –DOX controls (100 relative units) were statistically significant (with only one exception: tyrosinase in lower DOX concentration in one cell line), as depicted directly in the Figure. With the exception of SK‐MEL‐3 cells where the changes were small, strong decrease in RNA levels for these differentiation markers was observed.

MITF transcriptionally up‐regulates dozens of downstream genes. Many of them are associated with the formation of the pigment melanin 35. Because melastatin, a MITF transcriptional target and a putative tumour suppressor, is sharply responding to MITF levels in melanocytes 36, we used real‐time PCR to estimate the mRNA levels of melastatin, together with determining the mRNA levels of the *bona fide* MITF target tyrosinase. Maintaining cells for only 4 days in DOX caused profound decreases in melastatin and tyrosinase in 5 cell lines, while only in SK‐MEL‐3 cells the changes were less pronounced but significant (Fig. 6A and B); this was possibly because the final MITF decrease was less dramatic compared to controls without DOX (Fig. 1B) in these cells. The antiapoptotic downstream MITF target livin has been also uniformly decreased in all cell lines, by Western blot (Fig. 3A). Together, differentiation has been reliably and quickly repressed by DOX‐dependent down‐regulation of MITF levels.

## Discussion

Epithelial to mesenchymal transition is a key process associated with the invasive and metastatic disease in epithelial cancers, and EMT‐like changes appear also during the phenotype switching in melanoma. Many genes change their expression during EMT‐like process. The most characteristic is the down‐regulation of E‐cadherin and up‐regulation of N‐cadherin, together with the activation of SNAIL (SNAI1) and ZEB1 expression. EMT‐like gene pattern has been induced in normal melanocytes by ectopic mutated BRAF 31. Several authors have reported that melanoma has slightly atypical profile of protein expression in EMT, as SLUG (SNAI2) and ZEB2 have been presented as pro‐differentiative genes coexpressed with high MITF, not participating in the EMT process 6, 29, 30, 37. The expression of EMT markers has been found to be highly heterogeneous with the predominant EMT signature being high‐N‐cadherin/high‐Axl/low‐MITF, whereas the differentiation pattern was characterized mostly by high‐E‐cadherin/high‐MITF in primary melanoma cell lines 38. Even individual cells in tumours have shown different expression patterns of EMT proteins with SLUG expression weakening during tumour progression 39. Our present results showed only two EMT‐specific changes, each in other cell line (Fig. 3B), whereas the presence or changes in other EMT markers were inconsistent after the reduction in MITF expression.

We also observed no change in expression of three SC markers. Only SOX2, the expression of which was shown to require the Hedgehog signalling in melanoma and is crucial for the self‐renewal and tumorigenicity of human melanoma‐initiating cells 34, sharply increased in three cell lines with induced low MITF. In the remaining three cell lines, SOX2 was not expressed (Fig. 3C). Although OCT4 was found earlier increased in siRNA‐MITF‐transfected SK‐MEL‐28 cells 33, we did not observe any OCT4 changes (Fig. 3C). This discrepancy might be explained by possible more efficient reduction in MITF using siRNA‐MITF. Formally, as MITF undergoes post‐translational modifications that might have modulated the effect caused by MITF decrease.

Intriguingly, the highly pro‐oncogenic and invasive Wnt/β‐signalling pathway has been found to be anti‐invasive in melanoma as β‐catenin blocks invasiveness 40. β‐catenin pathway acts upstream of MITF and activates its transcription, and high‐MITF levels are anti‐invasive. MITF also suppresses the Rho‐GTPase‐regulated invading and interferes with β‐catenin‐induced expression of the pro‐invasive enzyme membrane type 1 matrix metalloproteinase 40.

Recently, two interesting studies which would at least partly explain the exclusive role of MITF in lowering invasiveness have implicated the expression of guanosine monophosphate reductase (GMPR), an enzyme of guanylate metabolism, in the regulation of invasiveness in melanoma cells. GMPR can deplete cellular GTP level, an event linked to lower melanoma invasiveness. The morphology of MITF‐depleted invasive cells is accompanied by a larger number of invadopodia 41. Subsequently, it has been shown that MITF is an upstream regulator of GMPR 42. Due to the lower GTP levels in cells overexpressing MITF or GMPR, the invasiveness would be suppressed. Oppositely, when siRNA‐mediated decrease in MITF was induced, with consequent declined levels of GMPR, even small increase in GTP (several per cents) generated high increase in invasion, which was even eightfold in 501mel cells and about twofold to threefold in SK‐MEL‐28 cells 42. High MITF concomitantly suppressed activity of RAC1, a kinase mutated in a subset of melanomas 43, and suppression of RAC1 activity was required to reduce invasiveness. Although we have also used clones from SK‐MEL‐28 and SK‐MEL‐5 cells displaying slower proliferation, no change in invasivity was recorded. Recently, glutamine depletion was shown to be sufficient to engender the decrease in MITF and invasiveness in melanoma cells. However, the MITF decrease could not be a cause of invasiveness, as glutamine starvation led to invasivity also in MITF‐negative cells 44. The transcription factor ATF4 alone down‐regulated MITF but surprisingly did not induce invasiveness. The authors suggested a mechanism of translation reprogramming whereby the eIF2B factor was found to be a crucial driver of melanoma invasiveness. Salubrinal, which inhibits dephosphorylation of complexes acting on p‐eIF2α, increased ATF4 and decreased MITF expression and induced invasiveness. Thus, as phosphorylated eIF2α inhibits eIF2B, this global reprogramming of translation involving high expression of ATF4 leads to invasiveness in melanoma cells 44. These findings where other factors besides sole MITF decrease are required to induce invasiveness are in conformity with the findings shown here.

The antiapoptotic role of MITF in melanomas is clearly established. However, some concern remains how the antiapoptotic signals are sustained in melanoma cell lines in which MITF expression is very low or in low‐MITF (and more invasive) areas of tumours. First, apparently, highly different cell context may exist among tumour cell subpopulations, and possibly single cells, that ensure antiapoptosis within the low‐MITF cells. Second, another one or more antiapoptotic genes, such as Axl or others ensure that low‐MITF cells do not undergo apoptosis. Previously, we have discussed whether so‐called ‘MITF‐negative’ melanomas are still melanomas, as they must have lost all MITF downstream differentiation markers 45. We argue that such cells either die due to the lack of MITF antiapoptotic function, as already documented in 501mel cells 27, or continue growing as an undifferentiated tumour if antiapoptosis is provided by other genes. The observed low‐MITF/high‐Axl populations in sections of human tumours 22 could serve as a possible example. What would be also conceivable is that in the course of cell line or tumour growth, cells might have adjusted MITF levels to amounts sufficient to promote proliferation, possibly with help of other pro‐proliferative (and antiapoptosis) protein(s), a notion that would reconcile both the rheostat model and our results as discussion above.

Inducibly and gradually decreased MITF level in melanoma cell lines, as described here, incurred slightly diminished proliferation, but the decrease was much smaller than anticipated taking into account the previous results 14, 18, 33. Low‐MITF populations such as some cell lines or slightly pigmented areas of tumours presumably utilize other proteins to maintain proliferation. We have observed various proliferation levels among isolated cell clones from the same cell line, indicating that even single cells in a relatively homogenous original cell line population may gradually create quite different proliferation potential when cultured longer under low‐MITF conditions; the growth of small number of clones almost halted proliferation while other clones proliferated at an unchanged rate (Fig. 4A and not shown). Consistent increase in the p27 protein, although slight, seems to be a more general hallmark of MITF down‐regulation. It is questionable whether the increase in p27 protein alone can incur the deceleration of proliferation in all types of low‐MITF cell lines and tumours subpopulations. Predictably, p27 protein might contribute to slow proliferation in some situations *in vitro* or *in vivo*. The *in vivo* effect of p27 has not been studied extensively.

The presented results bring more complexity to the phenotype switching process with the emphasis on the cell context and individual levels of MITF in cell lines and possibly even in single cells. It is highly probable that the primary functions of MITF in melanoma are to maintain the lineage identity (by regulating the downstream differentiation markers) and to play the indisputable antiapoptotic role. We further suggest that diminution of MITF level may accompany rather than induce the invasive phenotype in tumours, and its lower level *in vivo* may then eventually participate in the slow proliferation of the invasive tumour subpopulations.

## Author contributions

J.V. and K.V. designed the study, cultured cells and carried out experiments; J.R. performed Western blots and most of other experiments; P.H. performed real‐time PCR and invasivity assays; K.V. and L.O. prepared the Figures and performed statistical analysis; and J.V. wrote the manuscript. All authors have read and approved the manuscript.

## Conflict of interest

The authors confirm that there is no conflict of interests.

## Supporting information


**Fig. S1** Complete blocking of MITF expression achieved by transfection of shRNA‐MITF cloned in pSUPER‐puro plasmid followed by a short 2 days puromycin selection.

**Fig. S2** Immunofluorescence with the anti‐MITF antibody confirming the knockdown of MITF.

**Fig. S3** Cell cycle profiles of cell lines grown with or without DOX.

**Fig. S4** Proliferation of long‐term cultures of cell lines in media with or without DOX.

**Fig. S5** Migration (wound‐healing assay) of six cell lines in – DOX and + DOX.

**Fig. S6** Viability of cell lines performed in the media with indicated concentrations of DOX.
