# Supplementary figures and images for: Inducibly decreased MITF levels do not affect proliferation and phenotype switching but reduce differentiation of melanoma cells
Source: J Cell Mol Med. 2018 Jan 25;22(4):2240–51. doi: 10.1111/jcmm.13506 (PMC5867098; doi:10.1111/jcmm.13506)

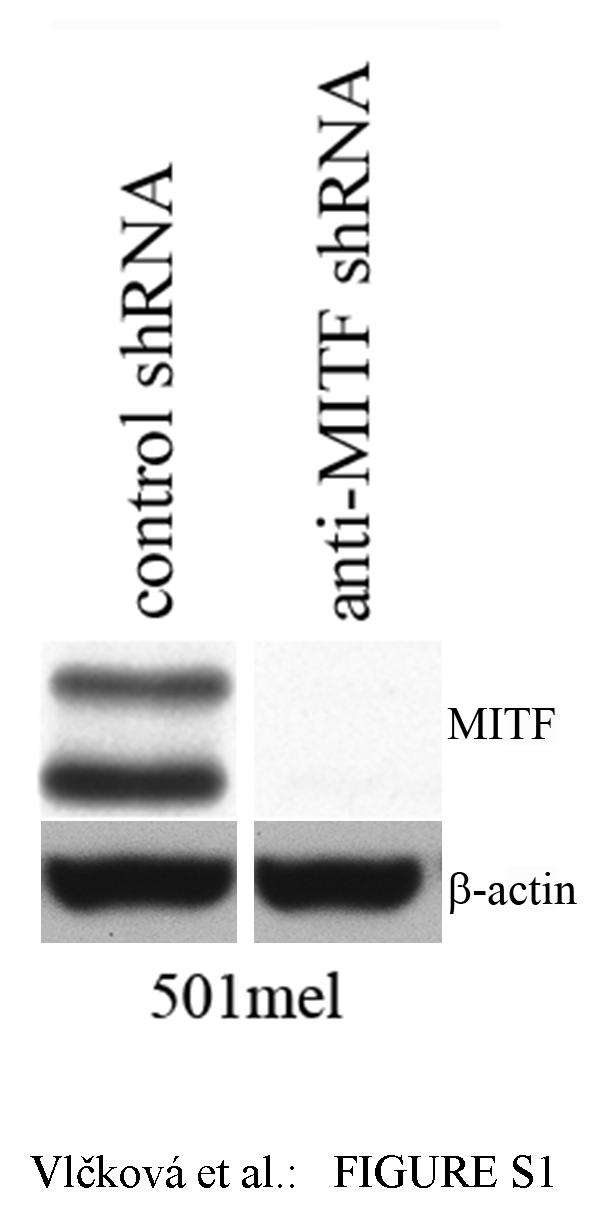

Supplement: Supplementary file 1 — Fig. S1 Complete blocking of MITF expression achieved by transfection of shRNA‐MITF cloned in pSUPER‐puro plasmid followed by a short 2 days puromycin selection. [file JCMM-22-2240-s001.tif]

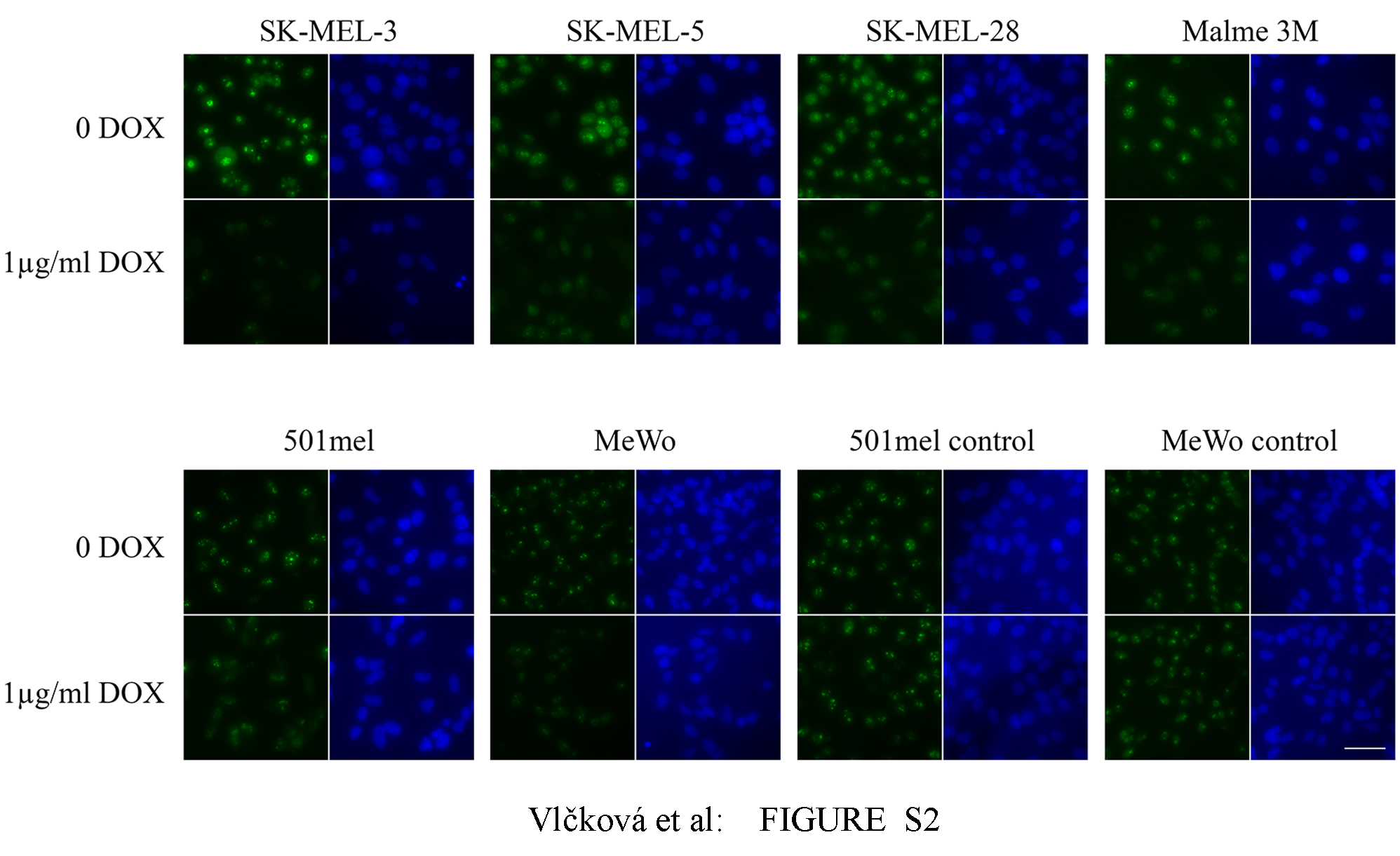

Supplement: Supplementary file 2 — Fig. S2 Immunofluorescence with the anti‐MITF antibody confirming the knockdown of MITF. [file JCMM-22-2240-s002.tif]

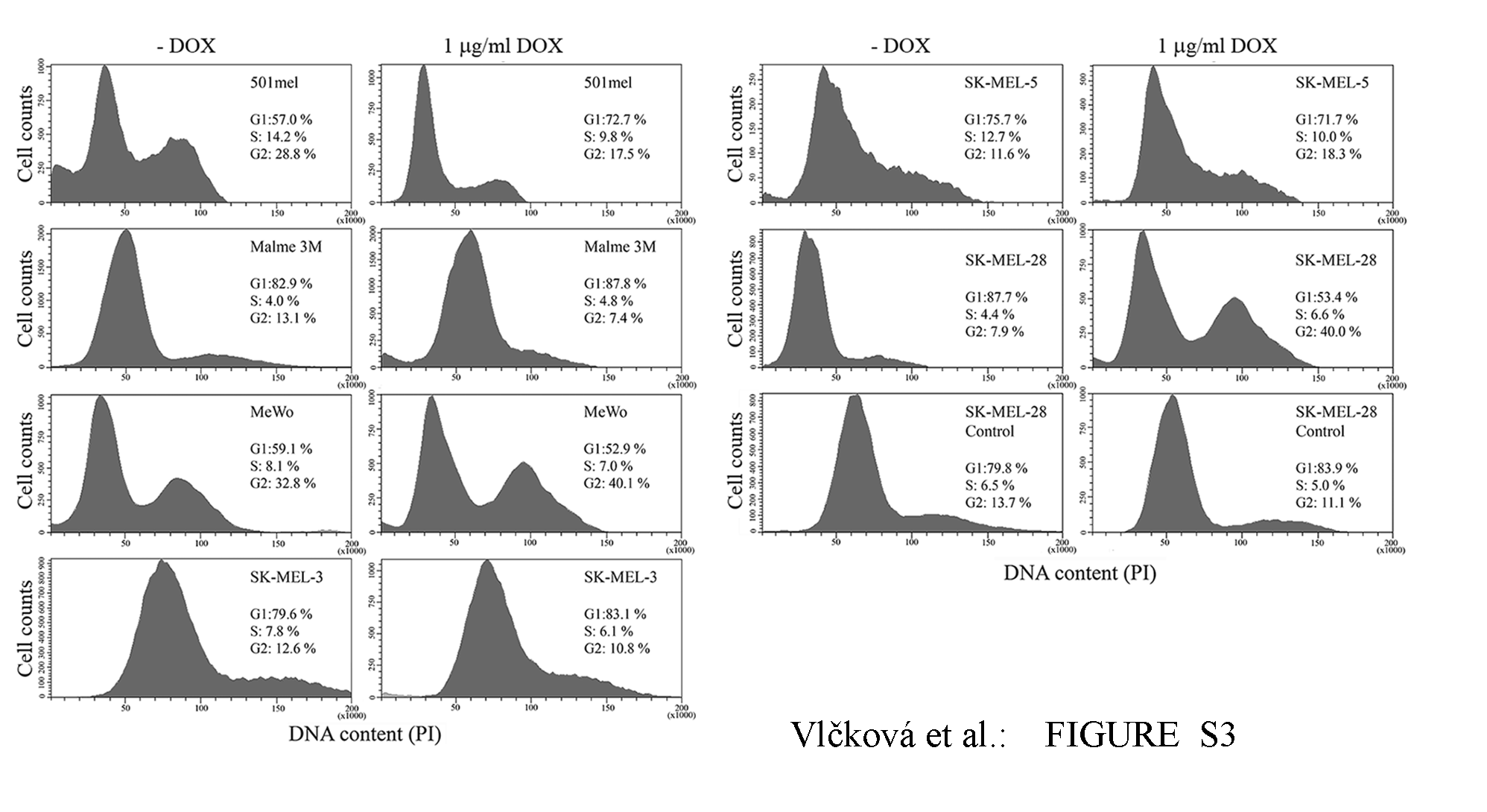

Supplement: Supplementary file 3 — Fig. S3 Cell cycle profiles of cell lines grown with or without DOX. [file JCMM-22-2240-s003.tif]

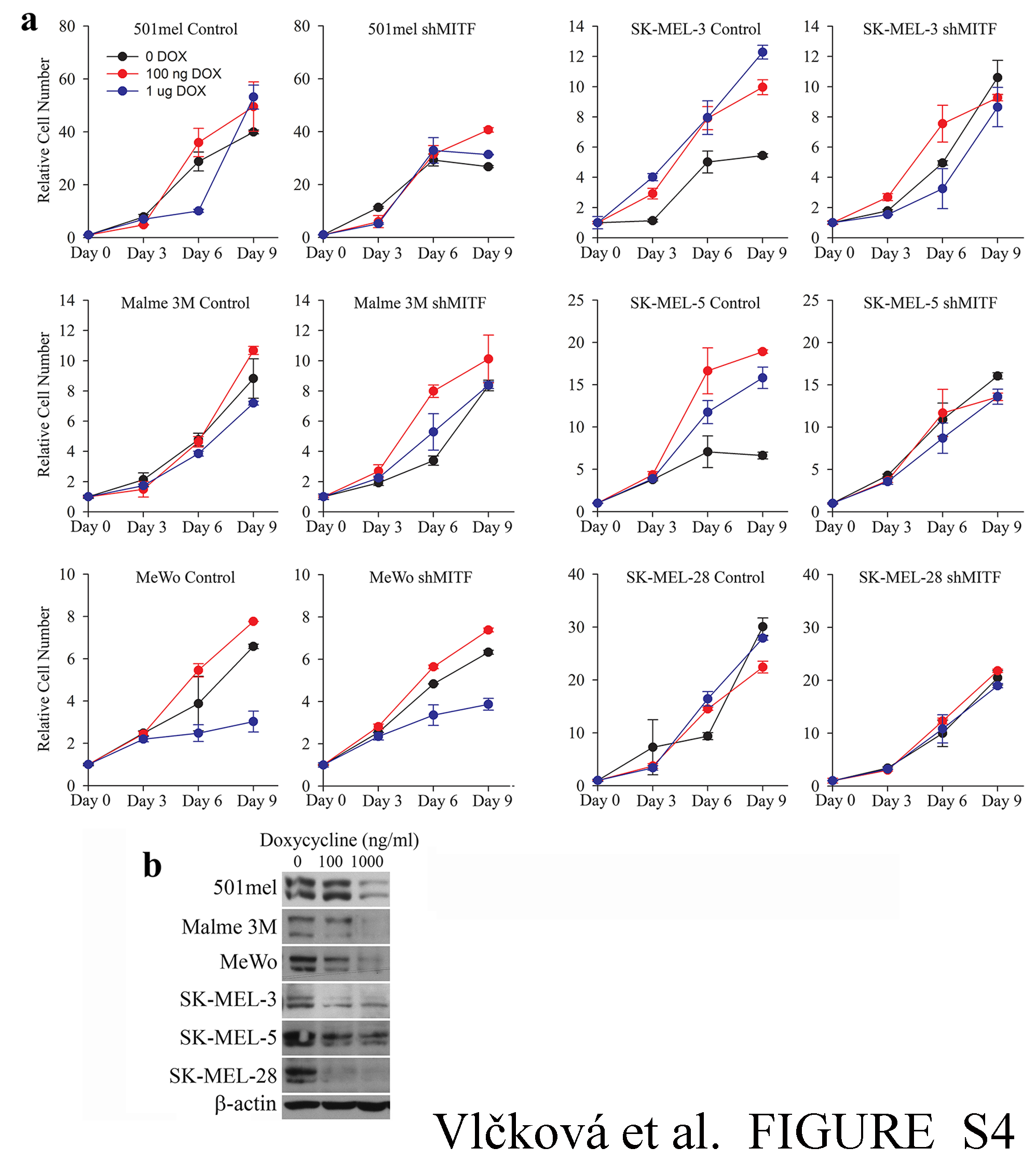

Supplement: Supplementary file 4 — Fig. S4 Proliferation of long‐term cultures of cell lines in media with or without DOX. [file JCMM-22-2240-s004.tif]

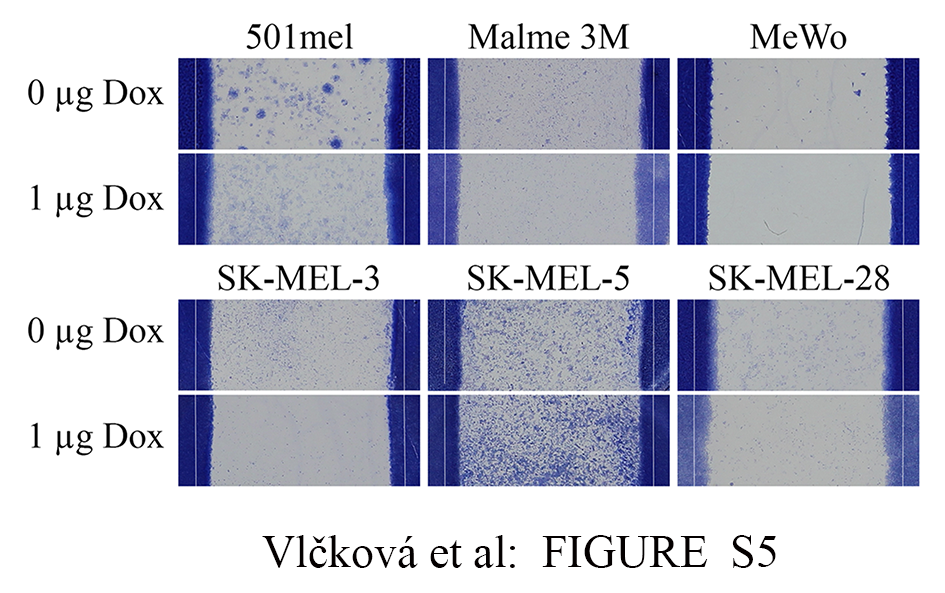

Supplement: Supplementary file 5 — Fig. S5 Migration (wound‐healing assay) of six cell lines in – DOX and + DOX. [file JCMM-22-2240-s005.tif]

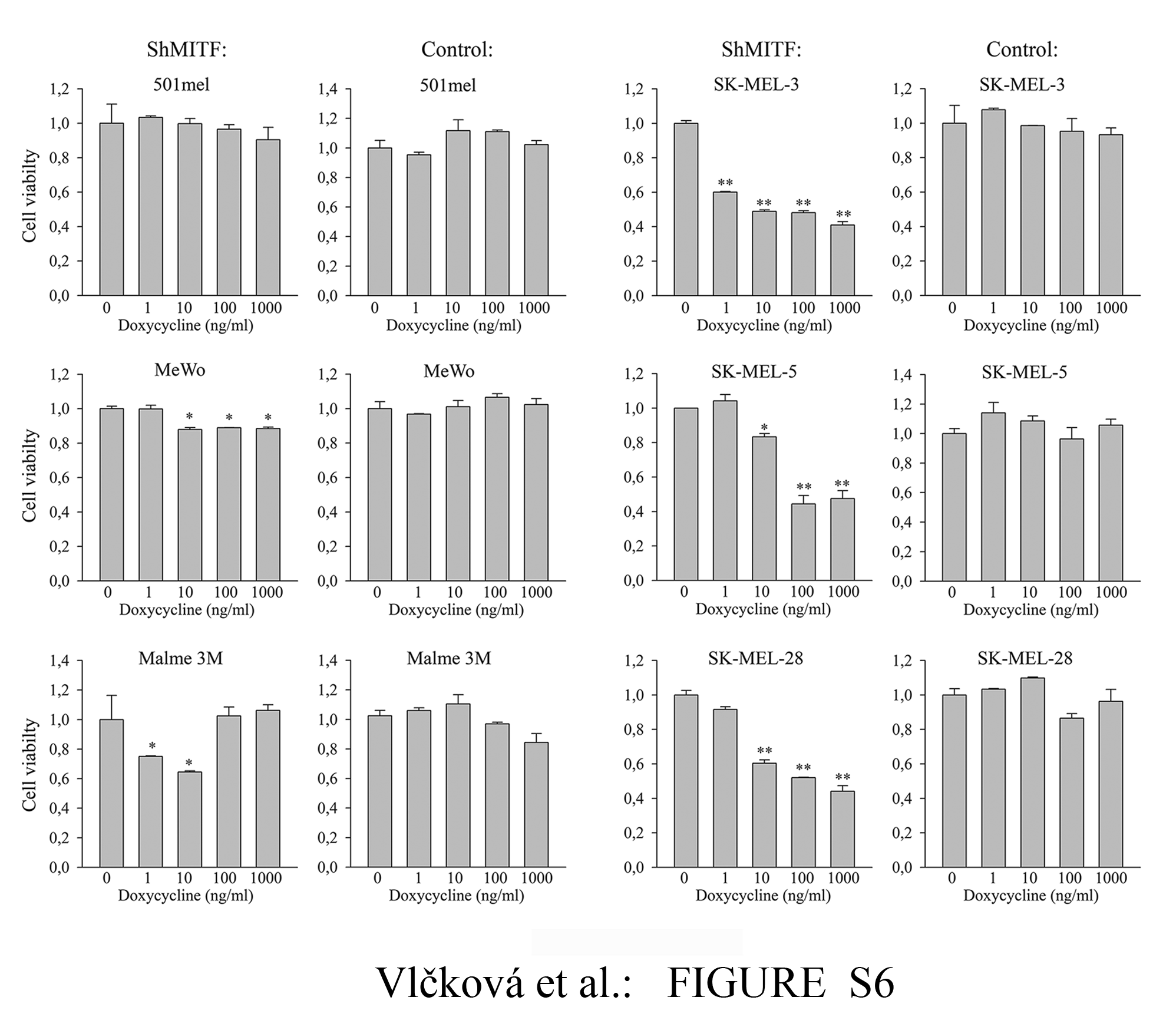

Supplement: Supplementary file 6 — Fig. S6 Viability of cell lines performed in the media with indicated concentrations of DOX. [file JCMM-22-2240-s006.tif]
