# Supplementary material for: Inducibly decreased MITF levels do not affect proliferation and phenotype switching but reduce differentiation of melanoma cells
Source: J Cell Mol Med. 2018 Jan 25;22(4):2240–51. doi: 10.1111/jcmm.13506 (PMC5867098; doi:10.1111/jcmm.13506)
Supplement: Supplementary file 7 [file JCMM-22-2240-s007.docx]

**Supplementary Figure Legends.**

**Fig. S1** Complete blocking of MITF expression achieved by a non-inducible transfection of shRNA-MITF used in this work. The target sequence was cloned in pSUPER-puro plasmid, transfected in 501mel cells, followed by a short 2 days puromycin selection. The RIPA extracts were prepared and Western blots performed. Control (scrambled) sequence did not have any effect on the MITF level. Actin has been used as a loading control confirming equal loading and the integrity of both samples.

**Fig. S2** Immunofluorescence with the anti-MITF antibody confirming the knockdown of MITF Immunofluorescence was performed with anti-MITF antibody (antibody dilution 1:200, left blocks) and DAPI (right blocks) in the identical image fields. Cells were left without DOX (upper blocks) or in 1 μg/ml DOX (lower blocks) for one week, replated into IF chambers and processed for IF next day. Scale bar 25 μm. Control cells (only two cell lines are shown as controls, similar results were obtained with the remaining four controls) do not show any difference when – DOX and + DOX fields are compared.

**Fig. S3**  Cell cycle profiles of cell lines grown with or without DOX. Cells were maintained in - DOX or + DOX media for one week and FACS profiles were then taken after staining DNA with propidium iodide. No appreciable changes were seen when – and + DOX cells were compared. Only in + DOX SK-MEL-28 cells the G2/S phase peak was (paradoxically) increased.

**Fig. S4** Proliferation of long-term cultures of cell lines in media with or without DOX. **(A)** Whole cell populations including control cells (containing scrambled shRNA) were cultured for five weeks in the indicated DOX concentration and then the proliferation rate experiment was performed in 24-well plates in triplicates. All cell lines grew normally during the five week period. **(B)** Control Western blot indicating the decrease of MITF was done before the experiment.

**Fig. S5** Migration (wound healing assay) of six cell lines in – DOX and + DOX. The migration of six cell lines was determined by the wound healing assay as described in Materials and Methods. No differences were observed between –DOX and + DOX cultures.

**Fig. S6** Viability of cell lines performed in the media with indicated concentrations of DOX. All cell populations (including controls) were cultured for 6 days in media without or with the indicated concentration of DOX. Next day, the viability assay was performed. Cells more sensitive to MITF decrease seem to be SK-MEL-3, SK-MEL-5, and SK-MEL-28. It is a result which roughly corresponds to the slightly lowered proliferation in these cells (see Fig. 1). Predictably, lowered viability was caused by increased apoptosis in cells requiring higher MITF for proliferation.
